# Safe and Ultraconservative Rehabilitation of Worn Teeth Patients: How Sectional Clear Aligners May Enhance the Prosthetic Treatment Plan

**DOI:** 10.1155/2022/8305893

**Published:** 2022-10-30

**Authors:** Camillo D'Arcangelo, Mirco Vadini, Matteo Buonvivere, Francesco De Angelis

**Affiliations:** Unit of Restorative Dentistry and Endodontics, Department of Medical, Oral and Biotechnological Science, School of Dentistry, “G. D'Annunzio” University of Chieti, Chieti, Italy

## Abstract

This study describes the clinical workflow for occlusal vertical dimension (OVD) increase in patients showing extensive tooth wear and mild teeth misalignment/crowding. A patient affected by dental erosion and occlusal abnormalities was treated to improve her situation. After ascertaining sound condyle and temporomandibular joint (TMJ) conditions, an OVD increase was sought to provide enough inter-occlusal space for the restorations. The use of TMJ three-dimensional imaging throughout the clinical procedures allowed to objectively track the condyle and disk position and confirm a steady condyle–glenoid cavity relationship before definitive restorations placement. Sectional clear aligner therapy prior to totally additive prosthetic rehabilitation allowed obtaining slight derotation and movements of anterior teeth, thus maximizing sound tissue preservation. Adhesively luted restorations were finally delivered on both anterior and posterior sectors. At the end of the treatment, the pre-operative TMJ balance appeared successfully preserved, and the patient was satisfied with the aesthetic and function achieved.

## 1. Introduction

Aesthetic concerns are nowadays part and parcel of every restorative and prosthetic rehabilitation: a satisfactory aesthetic result is as considerable as a proper functional outcome or a minimally invasive approach.

Simple cases involving frontal teeth aesthetics in the presence of balanced occlusion and proper inter-occlusal space are part of routine clinical practice [1, 2]. Situations involving generalized tooth wear require instead a more comprehensive approach: occlusal vertical dimension (OVD) increase is, in fact, frequently needed to obtain aesthetic improvement in such cases [3–6]. However, the debate regarding the most suitable method to safely increase OVD is still ongoing in the literature [7–20]. Over the last years, the introduction of accurate three-dimensional imaging exams, such as magnetic resonance imaging (MRI) or cone beam computed tomography (CBCT), gave to the clinician an objective tool to take under control the position of the temporomandibular joint (TMJ) during OVD increase.

Additive adhesive dentistry allows to get the most out of OVD increase. Without the need to seek mechanical retention with traditional preparation designs, in fact, sound cervical tooth structure can be saved [1, 2, 21], thus completely preserving the already worn teeth. When tooth wear is coupled with teeth crowding/misalignment, however, an orthodontic treatment with braces or aligners should be performed prior to the prosthetic phase aiming at straightening the teeth, thus providing spaces for minimally invasive intervention. In case of slight misalignment confined to the anterior sector and when posterior teeth do not need to be subjected to any movement, a sectional clear aligner (SCA) therapy might be indicated [22].

In the present clinical case, a safe clinical workflow optimized to aesthetically restore the smile of an asymptomatic patient affected by tooth wear and mild teeth misalignment is described. Adhesive and totally additive lithium disilicate indirect restorations were used in combination with SCAs, to preserve all residual sound tissues and to be as much conservative as possible. An MRI-based diagnostic approach was employed to evaluate the condyle–fossa relationship and keep it unchanged throughout the whole treatment.

## 2. Case Report

**Figure 1:**
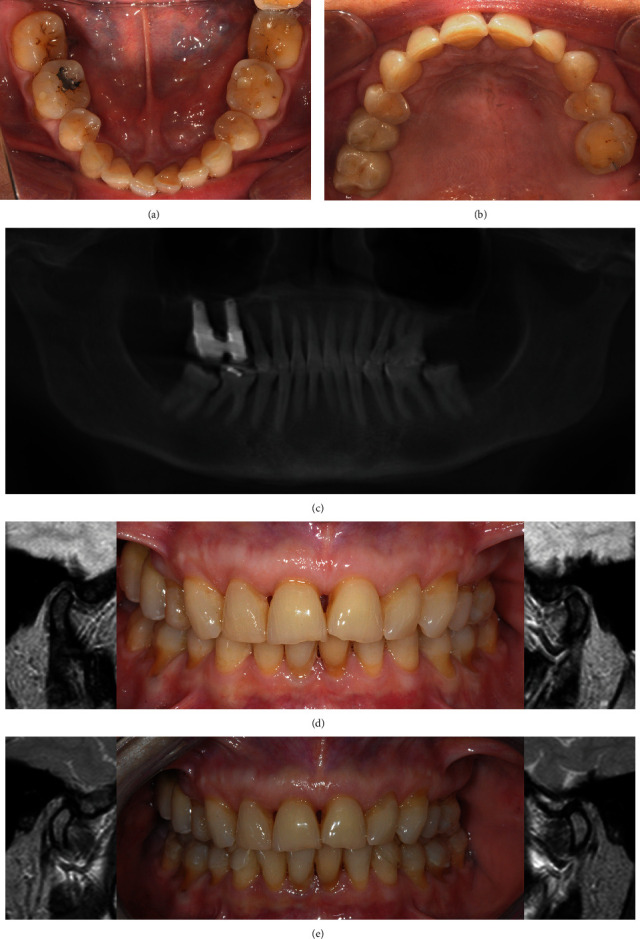
Clinical workflow for an occlusal vertical dimension increase in a patient showing worn dentition. ((a) and (b)) Pre-operative intra-oral occlusal photographs showing signs of cervical abrasion/erosion, slight misalignment in the upper anterior, and mild crowding in the lower-anterior areas: aesthetics appears negatively affected. (c) Panoramic X-ray image showing the pre-operative initial condition. (d) A pre-operative temporomandibular joint (TMJ MRI) scan was taken, with the patient in maximum intercuspation, showing healthy and balanced condyle–disc–fossa relationship both on the left and right side. (e) A second TMJ MRI scan was taken with patient dressing resin posterior fixed splints in order to check the TMJ status in the new mandibular position.

A 65-year-old woman with unremarkable medical history was referred with a chief complaint concerning the unsatisfactory aesthetic of her smile. The intra-oral examination revealed extensive tooth wear involving anterior and posterior teeth on both upper and lower arches, signs of cervical abrasion/erosion, slight misalignment in the upper anterior, and mild crowding in the lower-anterior areas (Figures 1(a) and 1(b)). As showed by the pre-operative panoramic X-ray image, teeth 1.7, 2.5, 2.7, 3.4, and 4.4 were missing, while teeth 1.5 and 1.6 had been previously replaced by implant-supported fixed dental prostheses (Figure 1(c)). Apart from teeth 3.6 and 4.6, which showed small amalgam and composite fillings, all the other elements did not display pre-existing restorations. All teeth were vital. The patient did not report symptoms of temporomandibular disorders (TMDs), and the clinical examination confirmed the absence of any dysfunctions affecting TMJ, masticatory muscles, and the associated structures. The proposed treatment plan consisted of a full mouth adhesive and additive rehabilitation in combination with the use of SCAs, aiming to improve the aesthetics with no tooth preparation, after having established a new and increased OVD.

The presence of physiological and healthy pre-operative TMJ conditions was objectified by MRI sequences taken in maximum intercuspidation using a 3 Tesla MRI system (Philips Ingenia 3.0 T, Philips Medical Systems, Best, The Netherlands) with 6-channel dS Flex M surface coil. *T*1-weighted sagittal sequences with a 2 mm slice thickness were obtained for each TMJ [6]. Both condyles appeared well centered within the glenoid cavities, in an anterior–superior position against the slope of the articular eminences (Figure 1(d)). On the basis of objective MRI analysis and due to the lack of TMD signs/symptoms or muscular contractions, the present preoperative condyle–fossa relationship was presumed to be sound. Thus, care was taken to keep the above-mentioned relationship unchanged throughout the treatment.

After placing a standard cheek/lip retractor, the patient was asked to perform a slight and slow mouth opening, starting from the maximum intercuspidation, up to a degree of tooth separation sufficient to assure enough inter-occlusal clearance for the restorative needs, trying to limit the OVD increase within the boundaries of pure condylar rotation. Any kind of operator-guided manipulation and/or bite registration medium in between the arches were avoided during this step of the procedure. Based on this, an OVD increase of 5 mm interincisally was supposed as appropriate. An indelible marker was used to mark the upper and lower canines and central incisors as references, so as to make the mandible positioning easily repeatable. After pushing two softened three-layer wax wafers (3 mm each) against the occlusal surfaces of left and right mandibular posterior teeth, the patient was asked to close while the operator guided the mandible in the new position, using the previously described marker signs on incisors and canines to prevent unsought excursive movements. The wax wafers were removed, cooled down, and placed back on the teeth to verify accuracy, stability, and fit.

The next step was aimed at objectively verifying that the new mandibular position, in the empirically proposed OVD, was truly within the limit of condylar pure rotation. For this purpose, self-curing low shrinkage acrylic pattern resin (Temp Red; Micerium S.p.A., Avegno, Italy) in dough stage was placed onto the upper anterior teeth before asking the patient to bite with the posterior wax wafers in place, thus obtaining an anterior jig. Following a facebow transfer, the jig, together with the wax wafers, served as inter-occlusal record to mount pre-operative casts on a semi-adjustable articulator (SAM 2P; SAM Präzisionstechnik GmbH, Gauting, Germany). On these casts, temporary and additive posterior splints extending from the second lower premolar to the second lower molar were manufactured with orthodontic acrylic resin (Orthocryl; Dentaurum) through salt-and-pepper technique. The splints extended bilaterally from the second lower premolar to the second lower molar and were fixed intra-orally on the unprepared teeth using a glass-ionomer cement (Ketac Cem; 3M ESPE). The patient was, then, subjected to a second TMJ MRI evaluation while dressing the posterior fixed splints, using the same parameters and the same corrected cross-sections described for the pre-operative exam. Comparing the two MRIs, the condylar position appeared substantially unchanged in respect to the articular fossa (Figure 1(e)), which confirmed that the proposed OVD increase could be safely considered within the limits of a pure rotation.

The posterior splints allowed for an additional diagnostic step, planned to clinically verify in a totally reversible way the real patient adaptation. After having dressed the fixed splints for two months, the patient reported a reassuring subjective adaptation to the new mandibular position, in the absence of TMD and muscle tenderness, which represented the required validation for the new proposed mandible position.

**Figure 2:**
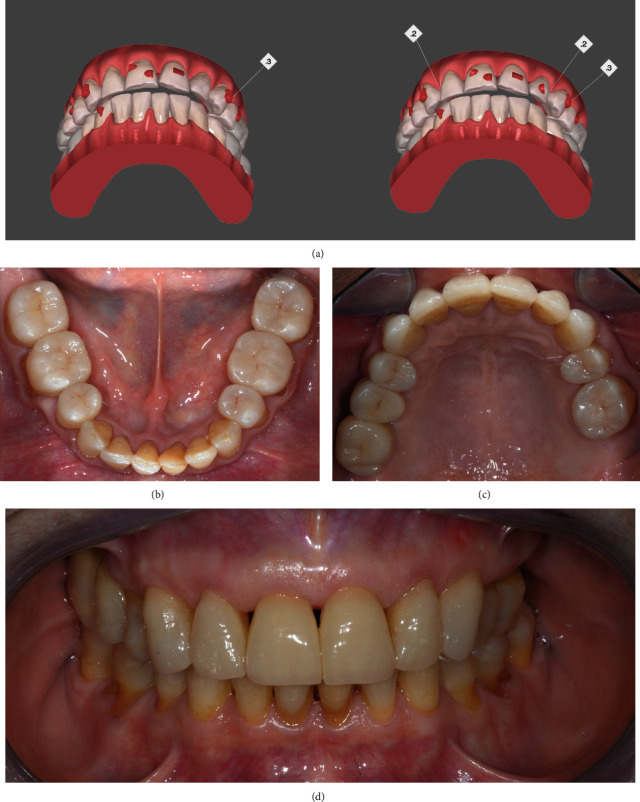
Sectional clear aligner therapy and final restorations. (a) 3D alignment simulation through ClinCheck software showing attachments bonded on teeth 1.1, 1.3, 1.4, 2.1, 2.2, 2.3, 2.4, and 4.3 and interproximal enamel reduction required on teeth 1.3, 2.3, 2.4, and 2.6. ((b)–(d)) Post-operative occlusal and frontal views. The main movements achieved after the clear aligner therapy were: derotation on teeth 1.1, 1.3, 1.4, 2.2, 2.3, and 4.3; tipping increase on tooth 2.1; torque increase on tooth 2.2.

The prosthetic finalization was performed adopting the splint-induced occlusal position as inter-maxillary relationship and took place starting from the left posterior sector. Left-side fixed splint was removed, providing access for restoring both upper and lower left premolars and molars. Sound posterior teeth were left unprepared. Old fillings were removed and replaced with direct composite (Enamel Plus BioFunction; Micerium S.p.A.) build-ups, and the residual sound tissues were not prepared. Polyvinylsiloxane impressions of the upper and lower arches were taken, holding the right-side fixed splint in place. Lithium disilicate (IPS e.max Press HT; Ivoclar Vivadent, Amherst, NY, USA) indirect overlays were manufactured using standard laboratory procedures, etched with 5% hydrofluoric acid, silanized, and adhesively luted: Enabond (Micerium S.p.A.) and warm Enamel Plus BioFunction composite were, respectively, used as adhesive and cement. Once stable and definite occlusal contacts were established on the left sector, the right-side fixed splint was removed, and both upper and lower right premolars and molars were restored as just described for the left side. Since the patient wanted to keep her pre-existing implant-supported fixed dental prostheses, lithium disilicate overlays were adhesively luted even on the old implant-supported ceramic crowns (teeth 1.5 and 1.6). At this stage, in order to close the residual open bite between upper and lower frontal teeth following the OVD incrementation, SCA therapy in both arches (Invisalign GO, Align Technology, Tempe, AZ, USA) was performed. A digital impression was acquired by means of an intra-oral scanner (iTero, Align Technology) and sent to the manufacturer for the 3D alignment simulation through the ClinCheck software (Align Technology; Figure 2(a)). After the approval, aligners were received, and attachments were bonded on teeth 1.1, 1.3, 1.4, 2.1, 2.2, 2.3, 2.4, and 4.3, respectively. Inter-proximal reduction was performed following the producer's recommendations: 0.2 mm of enamel was removed from both the mesial and distal sides of tooth 1.3; 0.2 mm from the mesial and 0.3 mm from the distal side of tooth 2.3; 0.2 mm from the distal side of tooth 2.4; and 0.2 mm from the mesial side of tooth 2.6. The therapy consisted of 15 aligners: the patient was instructed to wear them at least 22 hours a day and to change them weekly; clinical control was repeated every 4 weeks throughout the treatment. At the end of the treatment, a derotation of teeth 1.1, 1.3, 1.4, 2.2, 2.3, and 4.3 was obtained, as well as a slight increase in tipping of tooth 2.1 and in torque of tooth 2.2. After the SCA therapy, the anterior teeth misalignment was corrected, and an anterior occlusal contact was re-established on natural enamel, leaving an appropriate overjet to safely restore the upper anterior teeth. Moreover, teeth position was optimized for the subsequent prosthetic phase. The six maxillary anterior teeth were restored using an indirect additive solution. New polyvinyl-siloxane impressions were taken, and the poured casts were placed on to the articulator to fabricate six no-prep lithium disilicate (IPS e.max Press HT; Ivoclar Vivadent) veneers. The veneers were etched using 5% hydrofluoric acid and silanized. Subsequently, they were adhesively luted, using Enabond (Micerium S.p.A.) as adhesive and pre-warmed with composite heating conditioner (Ena Heat; Micerium S.p.A.) Enamel Plus BioFunction (Micerium S.p.A.) as cement. A life-long retention protocol (Vivera Retainer, Align Technology) was prescribed to stabilize teeth position achieved after SCA therapy.

After one week, the patient was recalled to collect post-operative clinical photographs (Figures 2(b), 2(c), and 2(d)). The patient aesthetic and functional needs appeared to be successfully satisfied.

## 3. Discussion

A full-mouth adhesive rehabilitation of a patient with no signs/symptoms of TMD, while showing generalized tooth wear and signs of malocclusion, was performed using a three-dimensional imaging to keep under control the TMJ conditions throughout the treatment. Totally additive prosthodontic procedures in combination with SCAs represented the ideal clinical choice to preserve all residual enamel and dentine structures.

The absence of pre-operative symptoms confirms that an OVD loss or a malocclusion is not always associated with a clinical TMJ impairment. When, in similar situations, a full-mouth rehabilitation is required for aesthetic purposes, it could be wise to limit the introduction of additional occlusal changes since they could become detrimental if the TMJ was just precariously balanced [23–27].

With the support of a solid MRI or CBCT validation of the proposed OVD increase, the use of temporary and additive fixed splints for two months provides a supplementary non-invasive diagnostic phase to rule out any risk of irremediable TMJ damage due to the abrupt occlusal change [23, 24, 27]. In this way, if any TMJ alteration or muscular abnormal response was identified during this reversible two-month-lasting step, the original occlusal conditions could be readily reestablished through the simple removal of the fixed splints. Among the multiple benefits offered by this clinical workflow, the prompt evidence of a potential condyle misplacement and the objective knowledge of the real jaw/condyle/disc positions prior to any permanent therapy seem to be of particular importance.

When dealing with full mouth rehabilitation cases, pre-prosthetic orthodontic treatment could represent a valid aid to achieve the desired aesthetical and functional outcomes. Correcting misalignment and crowding and closing open bite before the prosthetic phase, in fact, allow to perform less invasive procedures, thus maximizing the preservation of tooth sound tissues. Nowadays, however, adult patients tend to reject the unaesthetic conventional labial fixed appliances. Lingual orthodontics could be an adequate alternative, but still shows some disadvantages, such as tongue discomfort, speech problems, and masticatory difficulties [28]. Moreover, both labial and lingual appliances require a well-trained orthodontist. In this light, newly developed SCAs [22] could constitute a valuable option to combine patient's demands with dentist's needs. Being limited to the aesthetic sectors, SCA therapy is perceived as quick and comfortable by the patients, while representing a valid tool for restorative dentists aiming at simplifying their prosthodontic procedures by treating cases with inter-disciplinary mindset [22].

## 4. Conclusion

Totally additive restorations in combination with SCAs appear to be the best solution in patients showing extensive tooth wear, with a large portion of sound tissues already lost. An OVD increase, adequately supported by TMJ three-dimensional imaging (MRI or CBCT), is always necessary to re-establish good aesthetics in these patients.

Paired with adhesively luted restorations, SCA therapy may represent an additional tool to approach these complex clinical cases in the least invasive way possible.

## Data Availability

Data supporting this research article are available from the corresponding author or first author on reasonable request.
